# Memory strategy use in older adults with subjective memory complaints

**DOI:** 10.1007/s40520-016-0635-1

**Published:** 2016-10-05

**Authors:** Nikita L. Frankenmolen, Eduard J. Overdorp, Luciano Fasotti, Jurgen A. H. R. Claassen, Roy P. C. Kessels, Joukje M. Oosterman

**Affiliations:** 1 Centre for Cognition, Donders Institute for Brain, Cognition and Behaviour, Radboud University Nijmegen, Montessorilaan 3, 6500 HE Nijmegen, The Netherlands; 2 Rehabilitation Centre Klimmendaal, Arnhem, The Netherlands; 3 Department of Medical Psychology, Gelre Medical Centre, Zutphen, The Netherlands; 4 Department of Geriatric Medicine, Radboud University Medical Center, Nijmegen, The Netherlands; 5 Department of Medical Psychology, Radboud University Medical Center, Nijmegen, The Netherlands

**Keywords:** Compensation strategies, Subjective cognitive complaints, Cognitive reserve, Ageing

## Abstract

**Background:**

Subjective memory complaints (SMC) are common among older adults, but it is unclear to what extent adults with SMC spontaneously use memory strategies to compensate for their memory problems. As SMC may be a risk factor for memory decline later, it is important to extend our knowledge about spontaneous compensatory mechanisms in older adults with SMC.

**Method:**

Self-reported strategy use and observed strategy use were assessed in 38 adults with and 38 without SMC.

**Results:**

Adults with SMC used more strategies in daily life than those without. In the SMC group, memory complaints were positively correlated with strategy use. Only in adults without SMC, a significant correlation was found between observed strategy use and task performance.

**Conclusion:**

Strategy use in older adults with SMC may be compensatory in nature, but did not increase their objective memory performance. Therefore, older adults with SMC might benefit from interventions aimed at optimizing strategy use.

## Introduction

Older adults with subjective memory complaints (SMC) experience a decline in memory functioning, without evidence for objective cognitive impairments or an underlying neurodegenerative disease or psychological disorder [1]. Although the experienced decline lies within the normal limits of cognitive ageing, it negatively influences everyday functioning and quality of life [2]. Moreover, older adults with SMC have an increased risk of future cognitive decline and dementia [3], which stresses the need for early intervention.

Many interventions targeting SMC incorporate strategy training aimed at compensating for the memory decline [4]. However, it is unclear to which extent older adults with SMC spontaneously use strategies to cope with their memory complaints. To improve interventions, it is important to gain more insight in spontaneous memory strategy use. Memory strategies can be divided into internal and external strategies [5]. Internal strategies include mental encoding or retrieval strategies, such as rehearsal or visual imagery. External strategies are memory aids supporting memory function, such as using a calendar or taking notes. Previous studies on healthy ageing have shown that it is specifically external strategy use that increases with age [6], presumably because the use of external aids requires little cognitive control and can easily be incorporated in daily life. In addition, adults with SMC recruit additional neural networks during memory encoding [7, 8], suggesting the use of compensatory cognitive functions. However, to date, it is unclear whether adults with SMC also use more strategies in daily life and to what extent spontaneous strategy use is associated with better memory performance on objective tests.

The present study examined memory strategy use in older adults with SMC and those without SMC. We expected both groups to use relatively more external than internal strategies [6] and expected older adults with SMC to use more strategies than those without [7, 8]. Furthermore, strategy use has been related to education level, premorbid IQ and memory performance [9–11]. Therefore, in both groups, correlation analyses were performed with these variables.

## Method

### Participants

The participants of this study comprised 38 older adults with subjective memory complaints (SMC) and 38 healthy older adults without SMC. All participants were aged between 50 and 85 and lived independently in the community. Exclusion criteria were severe psychiatric problems, neurological disorders, substance abuse and objective cognitive impairment. The participants with SMC were recruited from three outpatient memory clinics in the Netherlands: Radboudumc Nijmegen, Canisius-Wilhelmina Hospital Nijmegen and Gelre Hospitals Zutphen, in the context of an ongoing study on the effects of a memory training. Patients were classified as having SMC by a multidisciplinary team of clinical specialists. Neuropsychological assessment was used as a supplementary tool to exclude clinical diagnoses such as mild cognitive impairment, dementia or psychiatric disorders. The older adults without SMC consisted of community-dwelling elderly who were recruited through advertisement and were screened over the telephone for exclusion criteria and for subjective memory complaints.

Table 1 shows the characteristics of the participants. Education level was rated according to the International Standard Classification of Education (ISCED) of the United Nations Educational, Scientific and Cultural Organisation Institute for Statistics (UNESCO-UIS). IQ was estimated using the Dutch version of the national adult reading test (NART). Memory complaints were assessed using a 30-item memory complaint inventory on which participants indicated the frequency of memory failures using a 5-point scale [12]. Inclusion of participants with SMC was part of a memory training study and was approved by the Medical Review Ethics Committee region Arnhem-Nijmegen (No. NL43519.091.013). Inclusion of the adults without SMC was approved by the Ethics Committee Faculty of Social Sciences (ECSS) at the Radboud University in Nijmegen.

**Table 1:** Descriptive characteristics of sample

Variable	Group	
	SMC−	SMC+
	*M* (SD)	*M* (SD)
*N*	38	38
Age	64.24 (8.92)	67.45 (7.40)
Sex M/F	20/18	18/20
Education	3.60 (2.01)	4.34 (1.89)
IQ estimate	106.84 (17.83)	106.00 (15.83)
Memory complaints	60.11 (13.58)	79.16 (14.94)
SOT story recall	8.95 (4.30)	8.36 (3.95)

*SMC*+ group with subjective memory complaints, *SMC−* group without subjective memory complaints, *SOT* strategy observation task

## Materials

### Strategy use inventory (SUI)

The strategy use inventory [13] measures strategy use in daily life with two subscales. The subscale External Strategies consists of 6 items and includes the use of memory aids, such as taking notes or using a calendar. The subscale Internal Strategies consists of 8 items and includes mnemonics such as mental rehearsal or creating associations. Participants had to indicate how often they use a certain strategy by rating items on a 5-point Likert scale. Average items scores were calculated for each subscale.

### Strategy observation task (SOT)

The strategy observation task (SOT) was developed in order to observe strategy use during a memory task that mimics daily life situations. Participants were instructed to remember a story (21 elements) that was presented as an audio clip. Story C of the Rivermead Behavioural Memory Test (RBMT) was used for this purpose. Additionally, participants were asked to spontaneously recall the story exactly 5 min after the end of the audio clip. In the meantime, they were instructed to solve basic math problems. The instructions emphasized that participants should remember as much information as possible and that they were allowed to do or use anything to help them remember the information. A pen, paper and an alarm clock were available within reaching distance. During the instruction, music was playing in the background. The volume of this music was preset at a level at which it was clearly distracting, but making sure that all instructions could still be understood. When asked, or at the start of the audio clip, the music was turned off. Strategic behaviour of the participants was recorded on an observation list. Examples of possible strategies were asking questions about instructions, asking to turn off the music, taking notes or setting an alarm. The total number of observed strategies was calculated for each participant.

The recall of the story ideally took place 5 min after the end of the audio clip. When participants did not recall the story spontaneously 7 min after the end of the audio clip, they received a cue. The SOT story recall score consisted of the number of correctly reproduced elements.

### Statistical analysis

For the statistical analyses, IBM SPSS 21.0 was used. Alpha was set at .05 for all analyses, one-tailed testing was used for directed hypotheses. Demographic variables of participants with and without SMC were compared using nonparametric, Chi-square or *t* tests. Subsequently, a 2 (group; between-subjects) × 2 (strategy type; within-subjects) mixed ANOVA was performed with ratio scores of the strategy use inventory as dependent variable. Furthermore, an univariate ANOVA was performed with group as independent variable and observed strategy use on the SOT as dependent variable. Finally, correlations were computed to examine whether education, IQ estimate, memory complaints and story recall correlated with strategy use in each group.

## Results

No between/group differences were found with respect to age [*t*(74) = −1.71, *p* = .092], sex distribution [*χ* ^2^(1) = .65, *p* = .819], education level (*U* = 890.50, *p* = .072), IQ estimate [*t*(74) = .22, *p* = .828] and SOT story recall [*t*(73) = .61, *p* = .544]. As expected, the SMC group reported significantly more memory complaints than the group without SMC [*t*(74) = −5.82, *p* < .001].

A significant main effect of strategy type [*F*(1, 74) = 18.41, *p* < .001, one-tailed] was found, in which the use of external strategies (*M* = 3.58, SD = .56) was more frequent than the use of internal strategies (*M* = 3.19, SD = .82) in both groups. The interaction between strategy type and group was not significant [*F*(1, 74) = .41, *p* = .523]. Furthermore, a main effect of group was found for strategy use [*F*(1, 74) = 3.67, *p* = .030, one-tailed], in which the SMC group (*M* = 3.51, SD = .64) reported using more strategies than the individuals without SMC (*M* = 3.26, SD = .72). Regarding the SOT, an ANOVA showed no main effect of group [*F*(1, 73) = .05, *p* = .411], indicating that both groups used the same amount of strategies during the SOT.

Table 2 shows the Spearman rank correlations between education, IQ estimate, EF, memory complaints, story recall and the strategy measures. In the SMC group, memory complaints were strongly associated with internal and external strategy use. Those who experienced more memory complaints also reported using more strategies in daily life. These correlations were not significant in the individuals without SMC. Education level and IQ did not correlate significantly with any of the strategy measures. The number of observed strategies during the SOT correlated positively with the story recall score in the individuals without SMC only; those who used more strategies had a better recall score.

**Table 2:** Spearman correlations between participant characteristics and strategy measures in older adults without (SMC−) and in older adults with subjective memory complaints (SMC+)

	Strategy measures					
	SMC−			SMC+		
	Internal	External	SOT	Internal	External	SOT
Education	.31	.20	.24	.29	.05	.12
IQ estimate	.28	.23	.06	.05	-.02	-.17
Memory complaints	.30	.19	.31	.54^***^	.50^**^	-.19
SOT story recall	.25	.24	.37^*^	.11	-.10	.23

*SOT* strategy observation task

* *p* < .05; ** *p* < .01; *** *p* < .001

## Discussion

This study examined memory strategy use in older adults with and without SMC. Both groups used more external than internal strategies in daily life, which is consistent with previous findings in ageing [6]. Furthermore, the older adults with SMC reported using more memory strategies in daily life than the individuals who did not experience SMC. Moreover, strong correlations were found between the amount of memory complaints and strategy use within the SMC group. Although none of the participants in the SMC group had any clinically relevant deficits as measured with neuropsychological tests, they may encounter problems in daily life tasks that require a complex interaction of cognitive skills [14]. The increased strategy use may reflect a compensatory approach, in line with recent evidence showing that participants with SMC recruit additional, compensatory neural networks for successful cognitive performance compared to older adults without SMC [7, 8].

This explanation, however, is at odds with the finding that observed strategy use was unrelated to the actual memory performance in the SMC group, in contrast to results in the group without SMC. Although further research is needed, it could be argued that older adults with SMC use strategies less efficiently than those without SMC. Previous results indeed showed that older adults with SMC benefitted less from a provided strategy during encoding than individuals without SMC [14], suggesting that they have difficulties implementing the provided strategy.

These findings may have consequences for interventions including strategy training. Since older adults with SMC already use more strategies in daily life, the utility of offering strategy training to people with SMC might be questioned. However, our results also show that the spontaneous increase in strategy use in itself does not ameliorate their complaints, possibly because strategy use in older adults with SMC is less efficient. An important aspect of older adults with SMC is that they often have an exaggerated memory-related achievement motivation and experience little control over their memory problems [15]. Therefore, interventions including expectancy modification and strategy training aimed at coping with memory problems in daily life could help older adults to optimize their strategy use and gain more control over their memory functioning. A limitation of previous intervention studies, however, is that none included measures of strategy use and subjective memory functioning in daily life [4], thereby lacking information on the efficacy of memory strategy training. Future studies should examine whether older adults with SMC benefit from such interventions and whether they are able to implement the strategies in daily life.

This study is the first to examine spontaneous strategy use in older adults with SMC. Measures included both self-reported strategy use in daily life and task-related strategy use using the SOT. However, the SOT was limited to observable, mostly external, strategies during a memory task. Future research should also include measures of various internal strategies during encoding, such as sentence generation or visual imagery [10], since these strategies are known to enhance task performance.

## Conclusion

This study shows that older adults with SMC may compensate for their experienced memory decline by using more compensatory strategies in daily life. However, increased strategy use did not correlate with performance on a memory task in adults with SMC, suggesting that the strategy use in older adults with SMC may not be as efficient as in older adults without SMC.

## References

[1] Jungwirth S, Fischer P, Weissgram S (2004). Subjective memory complaints and objective memory impairment in the Vienna-Transdanube aging community. J Am Geriatr Soc.

[2] Montejo P, Montenegro M, Fernandez MA (2011). Subjective memory complaints in the elderly: prevalence and influence of temporal orientation, depression and quality of life in a population-based study in the city of Madrid. Aging Ment Health.

[3] Luck T, Luppa M, Matschinger H (2015). Incident subjective memory complaints and the risk of subsequent dementia. Acta Psychiatr Scand.

[4] Metternich B, Kosch D, Kriston L (2010). The effects of nonpharmacological interventions on subjective memory complaints: a systematic review and meta-analysis. Psychother Psychosom.

[5] Dixon RA, Hultsch DF (1983). Structure and development of metamemory in adulthood. J Gerontol.

[6] Bouazzaoui B, Isingrini M, Fay S (2010). Aging and self-reported internal and external memory strategy uses: the role of executive functioning. Acta Psychol.

[7] Erk S, Spottke A, Meisen A (2011). Evidence of neuronal compensation during episodic memory in subjective memory impairment. Arch Gen Psychiatr.

[8] Rodda JE, Dannhauser TM, Cutinha DJ (2009). Subjective cognitive impairment: increased prefrontal cortex activation compared to controls during an encoding task. Int J Geriatr Psychiatry.

[9] Barulli DJ, Rakitin BC, Lemaire P (2013). The influence of cognitive reserve on strategy selection in normal aging. J Int Neuropsychol Soc.

[10] Dunlosky J, Hertzog C (1998). Aging and deficits in associative memory: what is the role of strategy production?. Psychol Aging.

[11] Frankenmolen NL, Altgassen M, Kessels R (2016). Intelligence moderates the benefits of strategy instructions on memory performance: an adult-lifespan examination. Neuropsychol Dev Cogn B Aging Neuropsychol Cogn.

[12] Deelman BG, Saan RJ, Deelman BG, Saan RJ, van Zomeren AH (1990). Memory deficits: assessment and recovery. Traumatic brain injury: clinical, social and rehabilitation aspects.

[13] Koning-Haanstra M, Berg IJ, Deelman BG, Deelman BG, Saan RJ, van Zomeren AH (1990). Training memory strategies: description of a method. Traumatic brain injury: clinical, social, and rehabilitational aspects.

[14] Pike KE, Zeneli A, Ong B (2015). Reduced benefit of memory elaboration in older adults with subjective memory decline. J Alzheimers Dis.

[15] Metternich B, Schmidtke K, Hull M (2009). How are memory complaints in functional memory disorder related to measures of affect, metamemory and cognition?. J Psychosom Res.

